# A systematic review of evidence on the association between hospitalisation for chronic disease related ambulatory care sensitive conditions and primary health care resourcing

**DOI:** 10.1186/1472-6963-13-336

**Published:** 2013-08-26

**Authors:** Odette R Gibson, Leonie Segal, Robyn A McDermott

**Affiliations:** 1 Health Economics and Social Policy Group, Division of Health Sciences, University of South Australia, Adelaide 5001, Australia; 2 South Australian Health and Medical Research Institute, Wardliparingga Aboriginal Research Unit, Adelaide 5001, Australia; 3 Sansom Institute, Division of Health Sciences, University of South Australia, Adelaide 5001, Australia

**Keywords:** Type 2 diabetes, Chronic disease, Primary health care resourcing, Ambulatory care sensitive conditions, Hospitalisation

## Abstract

**Background:**

Primary health care is recognised as an integral part of a country’s health care system. Measuring hospitalisations, that could potentially be avoided with high quality and accessible primary care, is one indicator of how well primary care services are performing. This review was interested in the association between chronic disease related hospitalisations and primary health care resourcing.

**Methods:**

Studies were included if peer reviewed, written in English, published between 2002 and 2012, modelled hospitalisation as a function of PHC resourcing and identified hospitalisations for type 2 diabetes as a study outcome measure. Access and use of PHC services were used as a proxy for PHC resourcing. Studies in populations with a predominant user pay system were excluded to eliminate patient financial barriers to PHC access and utilisation. Articles were systematically excluded based on the inclusion criteria, to arrive at the final set of studies for review.

**Results:**

The search strategy identified 1778 potential articles using EconLit, Medline and Google Scholar databases. Ten articles met the inclusion criteria and were subject to review. PHC resources were quantified by workforce (either medical or nursing) numbers, number of primary care episodes, service availability (e.g. operating hours), primary care practice size (e.g. single or group practitioner practice—a larger practice has more care disciplines onsite), or financial incentive to improve quality of diabetes care. The association between medical workforce numbers and ACSC hospitalisations was mixed. Four of six studies found that less patients per doctor was significantly associated with a decrease in ambulatory care sensitive hospitalisations, one study found the opposite and one study did not find a significant association between the two. When results were categorised by PHC access (e.g. GPs/capita, range of services) and use (e.g. n out-patient visits), better access to quality PHC resulted in fewer ACSC hospitalisations. This finding remained when only studies that adjusted for health status were categorised. Financial incentives to improve the quality of diabetes care were associated with less ACSC hospitalisations, reported in one study.

**Conclusion:**

Seven of 12 measures of the relationship between PHC resourcing and ACSC hospitalisations had a significant inverse association. As a collective body of evidence the studies provide inconclusive support that more PHC resourcing is associated with reduced hospitalisation for ACSC. Characteristics of improved or increased PHC access showed inverse significant associations with fewer ACSC hospitalisations after adjustment for health status. The varied measures of hospitalisation, PHC resourcing, and health status may contribute to inconsistent findings among studies and make it difficult to interpret findings.

## Background

Primary health care (PHC) has been established as an integral part of a country’s health care system [1]. Key functions of PHC as described in the *Ottawa Charter*[2] include: keeping people and populations healthy; free from contractible illness and disease; providing timely treatment of treatable acute episodes of illness; and managing existing health conditions, in particular chronic conditions.

It is well documented that the natural progression of many chronic conditions results in multiple and complex morbidity [3]. When chronic disease is managed well disease progression may be slowed and further morbidity prevented [4]. Managing chronic diseases in the PHC setting is promoted through the use of evidence-based care guidelines that require a multi-disciplinary team approach to care [5]. For this reason, the Australian Government provides monetary incentives to general practitioners (GPs) to prepare a care plan for each of their patients with a chronic illness [6].

To evaluate how well a PHC service is performing, hospitalisations are identified that could be avoided with accessible and high quality PHC. Such hospitalisations are commonly termed Ambulatory Care Sensitive Conditions (ACSC) or avoidable hospitalisations. The initial set of ACSC were defined in the United States in 1976 and has since been used as indicators of access to and the performance of PHC in Australia and elsewhere [7,8].

Subsequently studies investigating access to PHC services, using ACSC as the outcome measure, were conducted in population groups facing different out-of-pocket costs in accessing PHC [9,10]. Weissman et al. [10] compared hospitalisation rates for ACSC in health care insured and uninsured populations and found uninsured patients had higher rates of ACSC than those with health insurance. The implication of what Weissman et al. [10] report in terms of ACSC in insured and uninsured patients is that removing financial barriers to access is important for better outcomes. It is recognised that even by removing the direct financial cost of care other barriers to accessing care may exist. Other barriers to accessing chronic disease management include lack of time to return to appointments, lack of health education and unavailable suitable transport [11]. In countries that have universal health care coverage, with subsidized or free access to PHC, such as in Australia, the United Kingdom and Spain, ACSC hospitalisation rates have been used primarily as an indicator of PHC quality (incorporating access) [12-14].

A set of ACSC has not been agreed upon universally. Generally though, the following categories of hospital conditions are considered to be ambulatory care sensitive; influenza and pneumonia, other vaccine preventable diseases, asthma, congestive heart failure, diabetes complications, chronic obstructive pulmonary disease, angina, iron deficiency anaemia, hypertension, nutritional deficiencies, dehydration and gastroenteritis, pyelonephritis, perforated/bleeding ulcer, cellulitis, pelvic inflammatory disease, ear nose and throat infections, dental conditions, convulsions and epilepsy, gangrene [15].

PHC is not the only determinant of an ambulatory care sensitive hospitalisation. In a review of the literature, Muenchberger and Kendall [16] identified significant predictors of avoidable hospitalisations for chronic diseases. The authors presented them in six categories (examples provided in brackets) as follows:

1. Symptom management (health status)

2. Supportive relationships (supportive spouse, house overcrowding)

3. Self-management support (personal resilience)

4. Coordination of care between primary, secondary and tertiary services (hospital discharge planning)

5. Local area liveability (air pollutants, geographical location)

6. Socio-economic opportunities (employment, health insurance)

The purpose of this review was to identify studies that investigated the association between diabetes-related hospitalisation and PHC resourcing. Diabetes-related hospitalisations typically represent the largest single category of ACSCs [17-22]. The specific questions of interest were:

● How were hospitalisation and PHC resource variables measured?

● Was the association between PHC resourcing and diabetes related hospitalisations significant, and if so, did the level of PHC resourcing increase or decrease hospitalisations?

● What patient health risks or community factors, like socio-economic status, were associated with diabetes-related hospitalisations?

## Methods

A search strategy was used to identify published studies that potentially addressed our research questions. Three database platforms were searched. EbscoHost platform was used to search the EconLit database. Search terms used were ‘hospital* OR ‘avoidable hospital*’ OR ‘ambulatory care sensitive’ AND ‘primary care’ OR ‘primary health care’ AND diabetes OR ‘type 2 diabetes’ OR ‘diabetes mellitus’. OvidSP platform was used to search the Medline database. Search terms were hospitalisation and primary health care and the Medline related words (for example hospitalisation, hospitalized) were applied. These were searched for in all fields (e.g. title, abstract) and across all geographic regions. Google scholar search engine was used with the term, ‘primary care resource and avoidable diabetes related hospitalisations’. The search was limited to English language, peer reviewed articles, and articles published between 2002 and 2012. References cited in articles considered eligible at the abstract stage were included in the review process.

### Inclusion criteria

There were four inclusion criteria:

1. Studies that used diabetes-related ACSC hospitalisations as at least one outcome measure of PHC performance or hospitalisation of persons with T2DM.

2. PHC resource variable(s) were quantified and included in the final model.

3. Studies in populations with a public health insurance scheme that financially covered all or the majority of health care costs. This was to exclude studies in populations where financial access barriers (high out-of-pocket costs for health care) would be a dominant influence on use of PHC and hospitalisations; given our research question was concerned with the impact of PHC resourcing in the context of universal health care access.

4. Studies published between 2002 and 2012, to ensure currency of health care systems and contemporary evidence for chronic care in PHC settings.

### Exclusion criteria

A systematic exclusion process was employed to arrive at the final set of review articles. From the initial search result article titles were reviewed. If it was obvious from the title that the study did not meet the inclusion criteria and was not peer reviewed (e.g. government report), the article was excluded.

Abstracts of articles not excluded by title were then reviewed against the inclusion criteria in addition to the following:

● no adjustment for confounders; individual or population health risk factors or community level characteristic that may affect hospitalisation, for example level of education

● duplicate articles

● articles that could not be retrieved.

Full text articles and their reason for exclusion are presented in Table 1.

**Table 1:** **Studies excluded at the full article review stage**^**a**^

**Exclusion criteria at article review**	**Reference**^**(notes for references)**^
Not a diabetes-related hospitalisation or a type 2 diabetes cohort	[23-29]
Did not measure a primary health care resource input	[30-33]^(31b)^
Did not adjust for patient level health risk of hospitalisation or social and economic factors that influence hospitalisation	[34-38]^(34c)^
Not a peer reviewed journal article	[39]
Analysis combining hospital outcomes and primary care inputs into a regional efficiency measure	[29]
Total articles excluded on full article review	18

Notes:

^a^ Some articles can be allocated against more than one exclusion criteria.

^b^ Identify number of GP visits per individual in describing the study population but do not include in multivariate analyses.

^c^ Adjusted for patient level health risks using all ACSC as the dependent variable.

### Data extraction

Data was extracted from the final set of articles and presented in tables.

Table 2 describes the diabetes-related hospital outcome measure and PHC resource inputs. For the hospital outcome measure this included noting the ACSC category (e.g. non-elective diabetes-related hospitalisations), reporting of results (e.g. hospitalisation rate), describing how hospitalisation was measured (e.g. rate per number of patients on the clinic diabetes register who had ≥ 1 hospitalisation within study timeframe), and the level of the hospitalisation measure (e.g. individual, facility, district area). The PHC resource variables were noted as described by the studies including their level of measure, for example, the number of patients per GP per facility. PHC resources significantly (p value ≤ 0.05) associated with the diabetes-related hospital outcomes were identified in a separate column to the non-significant PHC resources. For the PHC resources that were significant it was noted whether they increased [↑] or decreased [↓] diabetes-related hospitalisations.

**Table 2:** Diabetes-related hospital outcome measures and primary health care resource inputs and direction of significant study findings

**First author, date published (country)**	**Hospital outcome measure**		**Primary health care resource inputs and direction of significant study findings**	
	**Category of diabetes-related ACSC (reporting of results)**	**Description of how measured (level of variable)**	**PHC resources significantly associated with an increase [↑] or decrease [↓] in hospitalisation**^**a**^ ** (level of variable)**	**PHC variables that were not significant i.e. p value >0.05, or reference measure (level of variable)**
Dusheiko 2011 (England)	Emergency (unplanned) hospitalisations due to (all) short-term diabetic complications^b^ (incidence rate)	Incidence rate per family practice (health centre) (f)	Nil	Population per FTE family physician (f)
Griffiths 2010 (England)	Non-elective diabetes-related hospitalisations (rate per facility)	Rate per number of patients on the register experiencing ≥ 1 hospitalisation (f)	Increase in the number of patients per FTE GP(f) [↓]^c^ <3038 patients per FTE practice nurse (f) [↑] 3039–3901 patients per FTE practice nurse (f) [↑] 4823–6210 patients per FTE practice nurse (f) [↓] 6210+ patients per FTE practice nurse (f) [↓]	Sole practitioner practice (f) Primary medical service contract (f) 3901–4823 patients per FTE practice nurse i.e. Quintile 3 (f)
		Rate per number of patients on the register experiencing ≥ 2 hospitalisation (f)	Number of patients per FTE GP (f) [↓] <3038 patients per FTE practice nurse (f) [↑] 3901–4823 patients per FTE practice nurse (f) [↑]	Sole practitioner practice (f) Primary medical service contract (f) 3039–3901 patients per FTE practice nurse i.e. Quintile 2 (f) 4823–6210 patients per FTE practice nurse (f) i.e. Quintile 4 6210+ patients per FTE practice nurse (f) i.e. Quintile 5
Lavoie 2010 (Canada)	Chronic ACSC hospitalisation (rate difference)	Average difference in rates of hospitalisation between level of primary care service^d^ (f)	Health centre versus no facility (f) [↓] Health office versus no facility (f) [↓] Health centre versus nursing station (f) [↑] Health office versus nursing station (f) [↑]	Nursing station and no facility (f)
Ng 2010 (Canada)	An acute hospitalisation for any reason among persons age 12 years or older with type 2 diabetes (odds ratio)	Status of hospitalisation (yes or no) (i)	An increase in self-reported number of GP contacts in the previous 12 months [↓]	Nil
Bruni 2009 (Italy)	Hyperglycemic emergency hospitalisations (probability of being hospitalised)	Hospitalised, yes or no (i)	As number of visits to diabetes outreach clinic increased (i) [↑] More patients per gp 1100–1500 and >1500 (iGP) [↑] Larger proportion of annual income from pay-for-participation (GP payments related to number of patients with diabetes) (iGP) [↓] Health district receives ≥75 % GP income from incentive schemes (d) [↓]	Patients per GP <1100 (ref) Practice type, i.e. sole practitioner (ref), association, network, group (iGP) Per cent diabetic patients (iGP) Per cent annual income pay-for-compliance (GP payments related to the number of quality improvement processes involved in e.g. diabetes audit) (iGP) Health district receives 25–75 % GP income from incentives schemes (d)
El-Din 2009 (Saudi Arabia)	Type 2 diabetes related hospitalisation (odds of being hospitalised)	Hospitalised, yes or no (i)	≥ 6 outpatient PHC clinic visits, except diabetes clinic (i) [↑]	No outpatient clinic visits (ref) (i) 1–5 outpatient clinic visits (i)
Lin 2009 (Taiwan)	Short-term diabetes ACSC and long-term ACSC modelled separately (relative risk ratio)	Status of hospitalisation (yes or no) (i)	More outpatient diabetes visits per year (i) [↑]	Diabetes management received (primary care clinic (ref), medical centre, regional or district hospitals (i)
Rizza 2007 (Italy)	Hospitalisation for diabetes ambulatory care sensitive conditions (odds ratio)	Status of hospitalisation (yes or no) (i)	As the number of patients per primary care physician increases (iGP) [↑]	Number of primary care physician visits in previous year (i) Number of specialist visits in community health services (f)
Gulliford 2004^e^ (England)	Hospitalisation for chronic conditions (chronic hospital admissions per 100 000 persons)	Rate of hospitalisation per 100 000 persons (ha)^f^	As GP supply increases per 10 000 weighted population (ha) [↓] As mean partnership size increases (ha) [↓] As proportion of sole provider practices increase (ha) [↑]	Per cent practices with diabetes service (ha)
Gulliford 2002^e^ (England)	Hospitalisation for chronic conditions (chronic hospital admissions per 100 000 persons)	Rate of hospitalisation per 100 000 persons (ha)	As GP supply increases per 10 000 persons (ha) [↓]	Nil

Notes:

^a^ Tested for inclusion in the final model with level of significance ≤ 0.05.

^b^ Authors also used acute, non-specific hyperglycemia, and hypoglycaemia as individual dependent variables (the all short-term diabetes complications shown in this table was the sum of each of these and the primary care resource variable is not statistically significant in any of the models).

^c^ Example of interpretation: the non-elective diabetes-related hospitalisation rate per facility decreases with an increase in the number of patients per GP; more patients per GP translate to less primary health care resources per capita.

^d^ Level of service includes: health office = part-time service, health centre = working hours limited and no after-hours care, nursing station = 24/7 care (including emergency).

^e^ Same data source.

^f^ Adjusted for confounders; deprivation score, proportion in semi or unskilled social class, proportion households with ethnic minority residents.

(i) individual level variable, (f) facility level variable, (d) district area level variable, (ha) health authority level variable, (iGP) individual GP level variable, [↑] result showed the PHC resource of interest increased hospitalisation, [↓] result showed the PHC resource of interest decreased hospitalisation, *ns* – Not significant, *(ref)* – Reference measure, *PHC* – Primary health care, *GP* – General practitioner, *FTE* – Full-time equivalent, *UK* – United Kingdom.

Table 3 identifies the type of analysis performed (e.g. stepwise logistic regression), how results were reported (e.g. risk ratio), type of study (e.g. longitudinal), and the variance explained by the final adjusted model (r-squared). All variables tested for inclusion in the final model were noted. For those variables that were significant, it was noted whether they increased [↑] or decreased [↓] diabetes-related hospitalisation.

**Table 3:** Description of the final model including diabetes-related hospitalisation predictor variables, other than primary health care resourcing

**First author and date published (country)**	**Type of analysis (reporting of results)**	**Study design (date)**	**Health risks and socio-economic factors**^**a**^**significantly associated with an increase [↑] or decrease [↓] in hospitalisation**^**b**^**(level of variable)**	**Independent variables that were not significant**^**c**^**or reference (level of variable)**	**Variance explained by the model (r-squared)**
Dusheiko 2011 (England)	Negative binomial regression (incident rate ratio)	Prospective open cohort (2001/02 to 2006/07)	% HbA1c ≤7.4/7.5 (f) [↓] % 7.4/7.5 < HbA1c ≤10 (f) [↓] % HbA1c monitored (f) [↑] Baseline hospitalisation rate (f) [↑] Average physician age (f) [↓] % non-principal [↓]physicians (f) Training practice (f) [↑] % females 15–44 & 75+ years (f) [↑] Diabetes prevalence (f) [↑] Mental health prevalence (f) [↑] Heart disease prevalence (f) [↓] COPD prevalence (f) [↑] Low income index (f) [↑] % smoking (c) [↑] % obese (c) [↑] % communal residents (c) [↓] Located urban sparse, village/hamlet, village/hamlet sparse [↓] Mean distance to nearest practice (c)[↑]	Practice population (f) Personal medical services contract practice(f) % female physicians (f) % UK qualified physicians (f) % males all age groups (f) % females by age group other than age 15–44 & 75+ years(f) % non-white (c) % incapacity benefit (c) % binge drinking (c)Education/qualification deprivation (c) Central heating deprivation (c) Crime (c) Urban location (ref) Located town and fringe and fringe sparse (f) Mean distance to nearest 5 hospitals (f)	Efron’s R^2 =^ 0.206
Griffiths 2010 (England) [outcome is ≥ 1 or ≥ 2 diabetes admissions]	Two-level multilevel model with GP practices nested within Primary Care Trusts (hospitalisation rate from count of admissions)	Cross sectional (2005/06)	Index of deprivation (f) [↑] % aged ≥65 years (f) [↓] % ethnic minority (f) [↓]	Least deprived (ref) Density (people per hectare) (f) GP ≥45 years (f) % female GPs (f) % GP qualified in UK (f)	Not reported
[outcome is standardised diabetes admission ratio]	As above	As above	Density (people per hectare) (f) [↑] Unadjusted T2DM prevalence (f) [↑] % female GP (f) [↓] % GPs UK qualified (f) [↓]	% ethnic minority (f) GP ≥45 years (f)	Not reported
Lavoie 2010 (Canada)	Generalised estimating equations (average difference in ACSC hospitalisation rates among different facility types)	Prospective open cohort (1984/85–2004/05)	Age group (f) [result not reported] Gender (f) [result not reported] Location (f) [result not reported]	Unknown	Not reported
Ng 2010 (Canada)	Multi-variate logistic regression (odds ratio)	Prospective cohort (2000/01 –2002/03)	Age ≥ 65 years (i) [↑] Female (i) [↓] Lower to middle household income (i) [↑] Health utility index^d^ (i) [↓] Other chronic conditions (i) [↑] Prior hospitalisations (i) [↑] Impact of health problems experienced often or sometimes (i) [↑] Physically inactive (i) [↑] Former or current smoker (i) [↑] Regular alcohol consumption (i) [↓] Current insulin use (i) [↑] ≥ 1 specialist consultations in past 12 months (i) [↑] Residing in high hospital use health region (i) [↑]	Age 12–44 years (ref) Age 45–64 years (i) Male (ref) Highest income (ref) Lower, middle, upper middle (based on quintiles) household income (i) Residence urban or rural (i) No other chronic conditions except diabetes (ref) No prior hospitalisation (ref), Impact of health problems never experienced (ref) Physically active (ref) Moderately active (i) Never smoked (ref) Occasional alcohol consumption (ref) Former or never consumed alcohol (i) Not currently on insulin (ref) Body mass index (i) Daily fruit and vegetable consumption (i) Unmet health care needs (i)	Not reported
Lin 2010 (Taiwan) [outcome is short-term diabetes ACSC]	Cox proportional hazard regression (relative risk of hospitalisation)	Prospective cohort (1997–2002)	New patient (i) [↓] Age (i) [↓] Age ≥60.5 years (i) [↑]	Existing patients (ref) Age <60.5 years (ref) Number of comorbidities (i) Medium continuity of care (i) Low continuity of care (i) Male (i)	Not reported
[outcome is long-term diabetes ACSC]	As above	As above	Medium (i) [↑] and low continuity of care (i) [↑] relative to high New patient (i) [↓] Age ≥60.5 years [↑]	High continuity (ref) Age (i) Number of comorbidities (i) Male	Not reported
Bruni 2009 (Italy)	Multi-level logit model (probability of being hospitalised)	Cross sectional (2003)	Age 65–75 years (i) [↓] Age >75 years (i) [↑] Insulin dependence (i) [↑] Male GP gender (i) [↑]	Age 35–65 years (ref) Gender (i) No insulin (ref) GP female (ref) GP age (i) Practice location rural (i) GP postgraduate qualifications (i) % diabetic patients (i) Endocrinology beds (d)	Not reported
El-Din 2009 (Saudi Arabia)	Stepwise logistic regression (odds of being hospitalised)	Case control	Gender (i) [↑] Presence of nephropathy (i) [↑] HbA1c ≥ 7 mmol/L (i) [↑]	Female (ref) Nephropathy not present (ref) HbA1c <7 mmol/l (ref)	Not reported
Rizza 2007 (Italy)	Multi-variate logistic regression (odds ratio)	Cross sectional (April–July 2005)	Number hospitalisations previous year (i) [↑]	Education level (i) Length of hospital stay (i) Self-reported health status (i) Sex (i)Age (i)	Not reported
Gulliford 2004 (England)	Multiple linear regression (chronic hospital admissions per 100 000 persons)	Cross sectional (1999)	% rural patients (ha) [↓] % GPs ≥ 61 years (ha) [↑] % practices with female GP (ha) [↓] % primary care clinic with contraceptive service (ha) [↓]	% patients >75 years (ha) % primary care services with child health surveillance services (ha)	Not reported
Gulliford 2002 (England)	As above	As above	Deprivation score^e^ (ha) Per cent households headed by semi or unskilled manual occupation (ha) Per cent with limiting long-term illness (ha)	Percent of households of ethnic minority (ha)	Not reported

Notes:

^a^ Increased prevalence or per cent unless otherwise stated.

^b^ Tested for inclusion in the final model with level of significance ≤ 0.05.

^c^ level of significance ≥ 0.05.

^d^ measured by health utility index mark 3 (HUI3).

^e^ based on proportion of people in a health authority who are unemployed, living in overcrowded accommodation, not in owner housing, and not owning a car (Townsend score).

(i) individual level variable; (f) facility level variable; (d) district area level variable; (ha) health authority level variable; (c) proportion of community or residents in the area; ns – not significant; (ref) reference; [↑] associated with an increase in the hospital outcome; [↓] associated with a decreases in the hospital outcome *GP* – General practitioner, *mmol/L* – Millimoles per litre, *COPD* – Chronic obstructive pulmonary disease.

The association between PHC resourcing and diabetes-related ambulatory care sensitive hospitalisation is reported for each study in Table 4 and whether or not the findings support the hypothesis that more PHC resources result in less hospitalisation.

**Table 4:** Core findings of the effect of primary care resourcing on diabetes-related ambulatory care sensitive hospitalisations

**First author, date**	**Primary health care resource variable**	**Direction of association with avoidable hospitalisations**^**a**^	**Supports hypothesis**^**b**^	**Comment**
Dusheiko 2011	GPs per population	Not significant	No	Adjusted for facility-level prevalence of diabetes, mental health conditions, heart disease
Griffiths 2010	Patients per GP	Significant inverse	No	No adjustment for clinical health risks
	Practice nurses per patient	Significant inverse	No	Adjusted for facility-level unadjusted diabetes prevalence
Lavoie 2010	PHC service availability (service categories: no permanent locally based service, part-time, working hours, 24/7 care)	Significant inverse	Yes	No adjustment for health status. Adjustment for age, gender and location but not reported
Ng 2010	GP contacts in the previous 12 months	Significant inverse	Yes	Adjusted for individual-level health utility, other chronic conditions, prior hospitalisations, lifestyle behaviours
Lin 2010	Diabetes outpatient visits	Significant positive	No	Adjusted for number of comorbidities and age
Bruni 2009	Use of diabetes outreach service	Significant positive	No	No adjustment for health status. Adjusted for age. Outreach service use was a proxy of disease severity.
	Patients per GP	Significant positive	Yes	
	Funding incentives to promote better quality care	Significant inverse	Yes	
El-Din 2009	≥6 PHC clinic visits	Significant positive	No	Adjusted for presence of nephropathy and HbA1c
Rizza 2007	Patients per GP	Significant positive	Yes	Adjusted for number of hospitalisations in previous year and length of stay and self-reported health status
Gulliford 2004, 2002	GPs per population	Significant inverse	Yes	No adjustment for health status.
	Partnership size	Significant inverse	Yes	Adjusted for proportion of patients per health authority with a limiting long-term illness. Partnership size is a proxy for better access to multi-disciplinary care team.

Notes:

^a^A positive association means the outcome and exposure variables increase or decrease in the same direction, i.e. more practice nurses per patient results in more hospitalisations. An inverse association means the outcome and exposure variables move in the opposite direction, i.e. more practice nurses per patient results in less hospitalisations.

^b^More primary health care resources are associated with lower hospitalisation.

## Results

Ten studies were identified that met the inclusion criteria and were included in this literature review.

**Figure 1:**
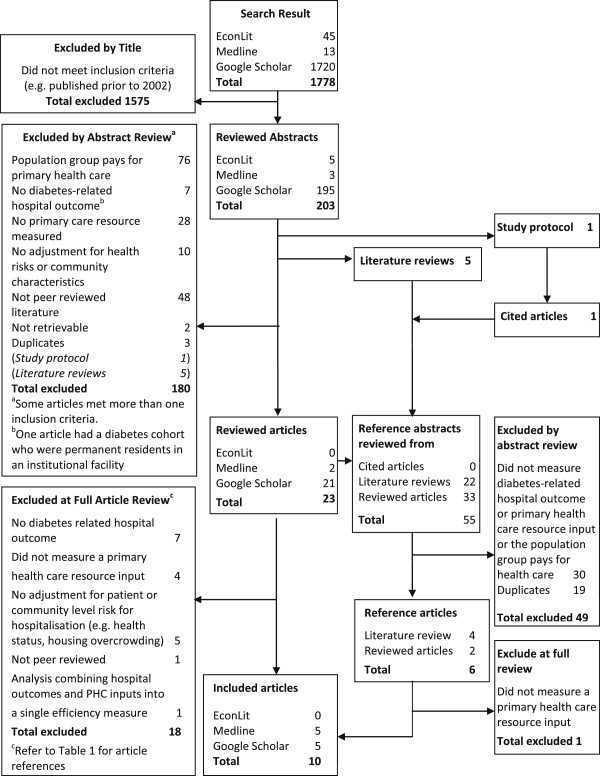
Flowchart of articles included and excluded by applying the search strategy.

The results of the search are detailed in Figure 1. The initial search retrieved 1778 articles. On review of their titles 1575 articles were excluded, leaving 203 abstracts. A review of the abstracts resulted in excluding a further 180 articles because they had an incorrect study population or did not measure a diabetes-related hospital outcome or a primary health care resource or were not peer-reviewed literature (Figure 1). This left 23 articles for a review of the manuscript, which led to a further 33 possible studies from reference checks, 19 of which were duplicates of those retrieved in the original search. On full review 18 of the 23 articles were excluded (Table 1). One article [29] that included relevant ACSC hospital outcomes and PHC resource inputs but combined the two into a global regional measure of efficiency was excluded on the basis that the results do not directly and specifically answer the review question which relates to the impact of PHC resourcing on diabetes-related ACSC hospitalisations. Five full review articles and two references met the inclusion criteria (n = 7). Five literature reviews produced 22 abstracts for review, three of which met the inclusion criteria. One study protocol was identified [40]. Google Scholar was used to identify cited articles. One article was found; however, it did not meet the inclusion criteria.

### Description of hospitalisation variable

Two measures of hospitalisation were used by all 10 studies (Table 2). The binary outcome; the absence or presence of a diabetes-related hospitalisation during the study period, at patient-level was used by half of the studies [19-22,41]. Half of the studies measured the rate of hospitalisation by facility or health district area level [17,18,42-44]. Lavoie and colleagues [44] measured the average difference in rates of hospitalisation for chronic conditions between communities with differing levels of PHC service resourcing (reflecting differential access to qualified health personal).

The category of diabetes-related ACSC differed among studies (Table 2), ranging from all diabetes-related ACSC, [18,20,22,41] to chronic ACSC [42-44] and only emergency [17,19] or acute or non-elective [18] hospitalisations related to diabetes. Lin and colleagues [21] analysed hospitalisations for short-term and long-term diabetes-related ACSC separately. Among the ten studies, three studies measured hospitalisation for the same ACSC (i.e. chronic conditions), [42-44] two studies measured diabetes-related hospitalisations [20,22] and the remaining five studies [17-19,21,41] each measured hospitalisation for a different set of ACSC.

### Description of primary health care resource variables

The measurement of PHC resources targeted five broad areas (Table 2):

1. Patient use of PHC services measured by the number of PHC clinic visits.

2. PHC workforce measured by FTE GP and/or nursing staff per facility.

3. Amount of PHC provided based on the service operating hours (e.g. provision of primary care several days a week or a 24-hour/7-days per week service).

4. Type of practice, for example, a sole GP service or a general practice group with a multi-disciplinary team.

5. Payment incentive schemes to improve PHC quality [19].

### Adjusters for confounding

Potential predictors of diabetes-related hospitalisations, other than PHC resourcing, included in models varied across studies and can be defined by the following broad categories. We note a number of these are attributes of the PHC system but not of resourcing:

1. Demographic characteristics of the individual patient or the PHC cohort or the catchment population that potentially use the PHC service.

2. Health status and/or lifestyle indicators of the individual patient, or the PHC cohort or the catchment population that potentially use the PHC service.

3. The geographic location of the service, whether rural or urban, as an indicator of access to PHC.

4. PHC provider characteristics defined by the personal profile of GPs such as age, gender and country of qualification, measured at the individual or facility level.

5. Social and economic characteristics of individual PHC patients or the PHC service catchment population.

PHC quality was measured by only one study, by the number of quality of care indicators delivered within the evidence based recommended time period. The variables used as potential adjusters for confounding in each of the ten studies are described in Table 3.

### Association between hospitalisation and level of primary health care resourcing

With the exception of Dushieko et al. [17] all studies found significant associations between avoidable diabetes-related hospitalisation and the level of PHC resourcing. These associations were not always inverse (i.e. more PHC resourcing fewer hospitalisations), some were positive (i.e. more PHC resourcing more hospitalisations) (Table 4). A summary of the findings on the association between hospitalisation and the level of PHC resourcing is provided below by category of the PHC resource measure.

#### PHC GP or nurse workforce FTE per capita

Four studies [19,22,42,43] reported that more GPs per capita or per n enrolled patients resulted in a decrease in the rate of avoidable hospitalisation for chronic conditions.

Griffiths et al. [18] was the only study, that included GPs/capita, to report a contrary finding. This study found that the rate of hospitalisation for non-elective diabetes-related hospitalisations decreased as the number of patients per FTE GP increased. Griffiths also reported the same pattern of a decreased rate of hospitalisation and increased number of patients per FTE with practice nurses. Hospitalisation was measured as the rate of registered patients per facility who experienced one or more diabetes-related hospitalisation [18].

#### Number of PHC visits

Three studies [19-21] found the probability of a diabetes-related hospitalisation increased as the number of PHC visits by the patient increased. One study [41] found that more self-reported visits to the GP in the 12 months previous to a diabetes-related hospitalisation resulted in less subsequent hospitalisation for the same reason.

#### PHC service availability

A crude measure of primary care service availability based on opening hours, identified that a more resourced service (e.g. PHC service available 24-hour/7-days per week compared to a service available 3 days/week) resulted in less hospitalisation for chronic conditions [44].

#### PHC practice size

Gulliford et al. [43] found that as resources in the primary care service increased, measured by mean partnership size, the hospitalisation rate decreased. Consistent with this was, as the proportion of sole practitioner PHC services within the health authority area increased hospitalisations increased.

#### PHC payment incentive schemes

Economic incentives for GPs to improve the quality of care to people with diabetes reduced the probability of hospitalisation [19].

## Discussion

Ten peer reviewed published studies of the association between ambulatory care sensitive diabetes-related hospitalisation and PHC resourcing were located through a rigorous search strategy.

Of the studies found, the measures of hospitalisation were limited to the dichotomous outcome of whether or not an individual experienced an avoidable diabetes-related hospitalisation or any chronic condition ACSC admissions during the study period, or measured the rate of hospitalisations. Other recognised ways of measuring hospitalisation such as cost of hospitalisation was not reported in any of the ten studies.

Even though the reason for hospitalisation was limited to diabetes-related or chronic condition ACSC, studies chose different ways to categorise the group of diagnoses (e.g. short and long-term diabetes-related ACSC) or chose a subset of diagnoses from this group (e.g. hyperglycemic emergency hospitalisations). Some of this variation may be explained by whether PHC access or quality was being evaluated and may reflect the complexity of PHC provision and T2DM disease pathways. For example the hospital outcome measure chosen by Dusheiko and colleagues [17] was unplanned emergency hospitalisations for short-term diabetes complications - their reason being that improved monitoring (i.e. improved quality of care) of patients with diabetes may increase elective admissions in the short to medium-term and in the longer-term reduce admissions for micro and macrovascular comorbidities [17].

The PHC resources measured also varied across studies and were used as proxy measures of PHC quality, availability or access. Reviewed studies identified the importance of including predictors of ACSC diabetes-related hospitalisations in the final model so that PHC resourcing was not wrongly attributed to the health outcome. Health status was included in seven of the ten studies (Griffiths et al. adjusted for health status when using standardised diabetes admission ratio as the outcome). Failing to adjust for health status is a weakness, given the known importance of health status for both hospitalisations and use of primary care services.

It is important to consider the complexities and limitations of using ACSC hospitalisations to measure the performance of PHC. By definition, primary health care is the first point of care that is continuous, coordinated and comprehensive whilst being accessible, acceptable and affordable to the population it serves [1]. The role of PHC is diverse and not simply about keeping people out of hospital. Therefore hospitalisation for ACSC can only ever be an incomplete and sometimes poor measure of the performance of PHC. The effect of PHC on ACSC was however the focus of this review, as an interesting policy question.

Much work has been done on rigorous selection of hospitalisations that would most likely be prevented with good ambulatory care [45-48]. Even so, the extent to which PHC can prevent or intervene in disease progression that may result in no or less hospitalisation (e.g. represented by decreased length of stay or a less severe reason for admission) will likely vary across conditions. The implication of this is that the impact of PHC on one ACSC hospitalisation is not uniform for each or across all ACSC hospitalisations. For example, a diagnosis of type 2 diabetes that occurs prior to related impaired kidney function (macroalbuminuria) will provide an opportunity for a comprehensive PHC service to prevent or slow progression to kidney disease. Whereas the same opportunity for PHC to intervene is lost if a diagnosis of diabetes is made, with already established renal impairment. This also highlights the importance of adjusting for individual disease stage [49] in statistical models.

Limitations of using ACSC hospitalisations to measure the performance of PHC also include those related to the measure of hospitalisation. Variation in hospital admission policies within and between hospitals and decisions made by hospital staff on the need to admit patients are likely to affect rates of hospitalisation for ACSCs [45]. It should also be noted that the quality of and access to PHC may influence some hospital admission policies and staff decisions on patient admission.

In addition, not all possible determinants of hospitalisation for chronic disease related ACSC, some of which were highlighted in the introduction, are accounted for in statistical models. This can distort the estimated impact of PHC on hospitalisation.

With due consideration of these complexities and limitations, ambulatory care sensitive hospitalisations are a useful measure of the performance of PHC at a population level, and of clear interest to policy makers. Hospital administrative data is objective, available and relatively inexpensive to gather. Gradual improvements in the scope and rigour of PHC data collection, that allows for more variables to be included in the models, should improve the accuracy and interpretability of the results of such studies.

The reviewed studies findings were mixed. Seven of the twelve PHC resource variables that had a statistically significant association with ambulatory care sensitive hospitalisations supported the hypothesis that more PHC resources are associated with less hospitalisation for ACSC. However, three of these studies did not adjust for health status [19,43,44]. Excluding the results of studies that did not adjust for health status, [19,43,44] six PHC resource variables remained and of these three supported the hypothesis that more PHC resources are associated with less hospitalisation for ACSC.

If all 12 significant findings are divided into two categories, by type of primary care variable, a clearer story emerges. Separating out i) the *use* of primary care services (e.g. n out-patient clinic visits) three of four reported relationships were positive - more visits were associated with higher rates of hospitalisation; from ii) *access* to primary care (eg GPs/capita, GP/patient list, range of PHC services) or incentives for higher quality of care; six of eight tested relationships (five of six studies) reported a significant inverse association between primary care and hospitalisation; that better access to quality primary care resulted in fewer ACSC hospitalisations. By applying the same categorisation (PHC *use* or *access*) to the six studies that adjust for health status, the conclusion remains that better access to primary health care resulted in fewer ACSC hospitalisations.

## Conclusion

There is little published evidence on the relationship between PHC resourcing and hospitalisation for diabetes-related ACSC. Studies use a range of measures of primary care and of hospitalisation, creating challenges for interpretation. Also the extent of adjustment for confounders is very mixed with three studies failing to adjust for health status.

While outcomes are mixed, in terms of the direction of the relationship, the impression from this body of work is that access to primary care (as distinct from use – which will be highly confounded by health status) is probably associated with a reduced rate of hospitalisation for diabetes-related ACSC.

Collectively, study findings must still be considered inconclusive, and the relationship between PHC resourcing and hospitalisation for diabetes-related ACSC remains uncertain. Thus additional studies are needed that adjust for a wide range of potential confounders and consider more carefully how best to adjust for disease severity.

## Abbreviations

ACSC: Ambulatory care sensitive conditions; PHC: Primary health care; GP: General practitioner; FTE: Full-time equivalent.

## Competing interests

The authors declare that they have no competing interests.

## Authors’ contributions

OG, RM, LS identified the literature review question. OG and RM devised the search strategy. OG performed the search strategy. OG systematically reviewed the articles against the inclusion and exclusion criteria collaborating with RM to arrive at the final 10 articles. OG and RM were primarily responsible for drafting the manuscript. LS contributed to discussion, critically reviewed and edited the manuscript. All authors read and approved the final manuscript.

## Pre-publication history

The pre-publication history for this paper can be accessed here:

http://www.biomedcentral.com/1472-6963/13/336/prepub
